# MTHFR gene variants and non-MALT lymphoma development in primary Sjogren’s syndrome

**DOI:** 10.1038/s41598-017-07347-w

**Published:** 2017-08-04

**Authors:** Sofia Fragkioudaki, Adrianos Nezos, Vassilis L. Souliotis, Ilenia Chatziandreou, Angelica A. Saetta, Nikolaos Drakoulis, Athanasios G. Tzioufas, Michael Voulgarelis, Petros P. Sfikakis, Michael Koutsilieris, Mary K. Crow, Haralampos M. Moutsopoulos, Clio P. Mavragani

**Affiliations:** 1 Department of Physiology, School of Medicine, National and Kapodistrian University of Athens, Athens, Greece; 2 Institute of Biology, Medicinal Chemistry and Biotechnology, National Hellenic Research Foundation, Athens, Greece; 3 Department of Pathology, School of Medicine, National and Kapodistrian University of Athens, Athens, Greece; 4 Research Group of Clinical Pharmacology and Pharmacogenomics, Faculty of Pharmacy, School of Health Sciences, National and Kapodistrian University of Athens, Athens, Greece; 5 Department of Pathophysiology, School of Medicine, National and Kapodistrian University of Athens, Athens, Greece; 6 Joint Academic Rheumatology Program, National and Kapodistrian University of Athens, School of Medicine, Athens, Greece; 7 First Department of Propaedeutic Internal Medicine, National and Kapodistrian University of Athens School of Medicine, Athens, Greece; 8 Mary Kirkland Center for Lupus Research, Hospital for Special Surgery, Weill Medical College of Cornell University, New York, NY USA

## Abstract

Primary Sjogren’s syndrome (pSS) confers increased risk for non-Hodgkin lymphoma (NHL) development. Two common polymorphisms, the c. 677C > T and c. 1298A > C, of the methylene-tetrahydrofolate reductase (MTHFR) gene, an enzyme essential in DNA synthesis and methylation, have been associated with susceptibility to NHL. Herein, we tested the hypothesis that MTHFR variants contribute to pSS-related lymphomagenesis. 356 pSS patients, of whom 75 had MALT and 19 non-MALT NHL and 600 healthy controls were genotyped for the detection of MTHFR polymorphisms. DNA methylation levels were assessed by pyrosequencing of the LINE-1 retroelement promoter in DNA from 55 salivary gland tissues from pSS patients. DNA double-strand breaks were determined in peripheral blood mononuclear cells from 13 pSS patients, using comet assay. Αnalysis according to lymphoma subtype revealed increased frequency of c. 677C > T TT genotype and T allele, as well as reduced prevalence of the c. 1298A > C C allele in the pSS non-MALT group compared to controls and patients without NHL. MTHFR c. 677C > T TT genotype was associated with reduced DNA methylation levels, while MTHFR c. 1298A > C AC genotype with reduced DNA double-strand breaks levels. MTHFR variants may be involved in SS non-MALT NHL development, through contribution to defective DNA methylation and genomic instability.

## Introduction

Primary Sjogren’s syndrome (pSS) is a chronic, systemic autoimmune disease affecting mainly the exocrine glands, resulting in oral and ocular dryness^1^. Among systemic autoimmune disorders, pSS has the highest susceptibility to non-Hodgkin’s lymphoma (NHL) development^2, 3^. The underlying pathogenic events leading from benign autoimmunity to malignant transformation remain elusive, although genetic contributors, such as mutations of the p53 gene and t(14:18) translocation, had been earlier proposed^4, 5^. While mucosa-associated lymphoid tissue (MALT) lymphoma is considered the main histological type of lymphoma encountered in the context of primary SS, diffuse large B-cell lymphoma (DLBCL) and follicular lymphoma (FL) may also occur^3, 6^.

A growing body of evidence over the last two decades consistently revealed features associated with immunecomplex formation -as a result of B-cell hyperactivity- as adverse predictors for the development of pSS related MALT lymphomas. These manifestations include among others the presence of palpable purpura, autoantibodies, C4 hypocomplementemia and monoclonal gammopathy^7–9^. Of interest, genetic aberrations affecting B-cell activation such as variants of the B-cell activating factor (BAFF) gene^10^, a recently described His159Tyr mutation of its receptor (BAFF-R)^11^, and a mutation of the tumor necrosis factor alpha-induced protein 3 (TNFAIP3) gene^12^ -an NF-κB pathway regulator- have been also associated with pSS MALT lymphomagenesis. In contrast, a recent study by Baimpa *et al*., suggested lymphocytopenia as an adverse predictor for SS related non-MALT lymphoma, mainly DLBCL^13^, with no associations between B-cell activation markers and non-MALT lymphoma development having been detected. These separate disease profiles suggest that distinct underlying molecular events are involved in pSS MALT and non-MALT NHL pathogenesis.

Chromosomal and genetic abnormalities are considered major underlying pathogenic mechanisms for NHL lymphomagenesis. Apart from genetic factors, epigenetic mechanisms, involving mainly methylation pathways, have been recently proposed as major contributors for both autoimmune disorders and NHL, particularly non-MALT subtypes^14–16^. Genetic variations of the methylene tetrahydrofolate reductase (MTHFR) enzyme -an enzyme essential in nucleotide synthesis and DNA methylation pathways- have been previously found to confer increased risk for both autoimmune disorders^17^ and NHL development^18–21^. However, no data regarding the role of MTHFR polymorphisms in pSS susceptibility have been reported so far.

MTHFR catalyzes the irreversible reduction of 5,10-methylenetetrahydrofolate (5,10-MTHF) to 5-methyltetrahydrofolate (5-MTHF), the dominant form of folate in the circulation, which subsequently through conversion to methionine, gives rise to S-adenosylmethionine (SAM) formation, the global methyl group donor, essential for the majority of biological methylation reactions. On the other hand, this process reduces the bioavailability of 5,10-MTHF. The latter ensures the conversion of nucleotides deoxyuridine-5-monophosphate to deoxythymidine-5-monophosphate (thymidylate) leading ultimately to decreased uracil levels. This is a critical step since elevated uracil levels significantly increase uracil misincorporation into DNA during replication, resulting in increased DNA double-strand breaks during the normal repair process. In contrast, dysfunction in the MTHFR enzyme can potentially lead to defective DNA methylation through reduction of SAM formation and, at the same time, to reduced DNA damage through decreased uracil levels. Single nucleotide polymorphisms of the MTHFR gene with clinical importance have been described, leading to decreased MTHFR activity through induction of amino acid changes: the c. 677C > T, p. Ala222Val and the c. 1298A > C, p. GLu429Ala^18, 22, 23^.

Herein, we tested the hypothesis that the MTHFR c. 677C > T and c. 1298A > C polymorphisms contribute to pSS pathogenesis and/or to pSS-related lymphomagenesis.

## Methods

### Study groups

The study included 262 pSS patients without lymphoma (pSS), 94 pSS patients diagnosed with lymphoma (pSS-lymphoma) and 600 apparently healthy controls (HC) of similar age and sex distribution. All patients fulfilled the revised international classification criteria for pSS^24^ and were followed up at the Rheumatology Department of General Hospital “G. Gennimatas”, the Institute of Autoimmune Diseases (Prof. HM Moutsopoulos) and the outpatient Clinic of the Department of Pathophysiology, University of Athens. The pSS-lymphoma group consisted of 75 patients with MALT (pSS MALT) and 19 with non-MALT (pSS non-MALT) lymphoma [12 DLBCL, 4 nodal marginal zone lymphoma (MZL), 2 small lymphocytic lymphoma (SLL) and 1 T-cell lymphoma]. Minor salivary gland (MSG) tissues from 55 pSS patients included in the initial cohort, as well as peripheral blood mononuclear cells (PBMCs) isolated from 13 pSS patients without lymphoma were implemented for methylation and comet assays, respectively (see below for further details). MSG tissues derived from 28 pSS patients uncomplicated by lymphoma and 27 diagnosed with NHL (23 MALT lymphoma, 1 DLBCL, 1 nodal MZL, 1 SLL and 1 T-cell lymphoma). Exclusion criteria for all groups were age younger than 18 years old and the presence of other systemic autoimmune disease. All groups were of Caucasian origin and had similar age and sex distribution. Blood samples were stored at −80 °C and processed in the Department of Physiology, National and Kapodistrian University of Athens. Informed consent was obtained from all study participants. The study protocol has been approved by the Ethics Committee of National and Kapodistrian University of Athens and all experiments were performed in accordance with relevant guidelines and regulations.

### MTHFR genotyping

Genomic DNA was extracted from blood samples, collected in EDTA tubes, using the Nucleospin Blood QuickPure kit (Macherey-Nagel GmbH & Co, Germany), according to the manufacturer’s instructions. DNA concentration was spectrophotometrically measured with Biospec-Nano (Shimadzu, Japan).

The c. 677C > T (rs1801133) polymorphism was analyzed by polymerase chain reaction (PCR) of genomic DNA using the following primer pairs: 5′-TGAAGGAGAAGGTGTCTGCGGGA-3′ (forward) and 5′-AGGACGGTGCGGTGAGAGTG-3′ (reverse). These primers generate a 198 bp fragment. PCR was carried out in a total volume of 15 μl with the Kapa Taq Ready Mix PCR Kit (Kapa Biosystems, Germany), containing 50 ng of genomic DNA and 0.2 μM of each primer. The PCR reaction profile was: initial denaturation at 95 °C for 3 min followed by 35 cycles including denaturation at 95 °C for 30 s, annealing at 58 °C for 30 s and extension at 72 °C for 30 s, with a final extension step at 72 °C for 3 min. PCR amplicons were digested for 2 hours at 37 °C with 10 units of the restriction enzyme HinfI (New England Biolabs). Then, the digestion products were electrophoresed on a 3% agarose gel, stained with ethidium bromide and visualized using ultraviolet illumination (Kodak EDAS). The wild type homozygous (CC), heterozygous (CT) and mutant homozygous (TT) genotypes produce one band of 198 bp, three bands of 198, 175 and 23 bp and two bands of 175 and 23 bp respectively, because the HinfI recognition sequence digests the 198 bp into 175 and 23 fragments.

The c. 1298A > C (rs1801131) polymorphism was determined using the following primer pairs: 5′-CTTTGGGGAGCTGAAGGACTACTAC-3′ (forward) and 5′-CACTTTGTGACCATTCCGGTTTG-3′ (reverse), using the same PCR conditions that were used for the C. 677C > T mutation. The amplified fragment of 163 bp was digested for 2 hours at 37 °C with 10 units of the restriction enzyme MboII (New England Biolabs). The c. 1298A > C abolishes an MboII restriction site. Digestion of the 163 bp fragment of the 1298AA genotype (normal) results in five fragments of 56, 31, 30, 28 and 18 bp, the 1298CC genotype (mutated) in four fragments of 84, 31, 30 and 18 bp, whereas the 1298AC genotype (heterozygous) in six fragments of 84, 56, 31, 30, 28 and 18 bp.

### Global DNA methylation status

To explore whether the presence of MTHFR variants is associated with alterations in global DNA methylation, methylation levels of CpG islands in the promoter of the long interspersed nuclear element 1 (LINE-1) were evaluated by pyrosequencing of genomic DNA derived from 55 minor salivary gland (MSG) tissues from pSS patients. LINE-1 elements have been used as an indicator of global DNA methylation status, because of their abundant presence in the genome of many eukaryotic organisms^25, 26^. A prevalidated pyrosequencing-based methylation assay was used to assess 4 CpG sites in the promoter region of LINE-1. PCR and pyrosequencing for LINE-1 methylation were performed using the Pyro-Mark kit (Qiagen, Valencia, CA). Each PCR mix contained the forward and reverse primer (each 0.4 μmol/L), 0.8 μmol/L of dNTPs, 1.5 mmol/L of MgCl_2_, 1x PCR buffer (Qiagen), 0.64 U of HotStart Taq polymerase (Qiagen), and 2 μl of bisulfited template DNA in a total volume of 20 μl. PCR conditions were as follows: initial denaturing at 95 °C for 15 minutes, 45 cycles of 95 °C for 20 seconds, 50 °C for 20 seconds, and 72 °C for 20 seconds, and final extension at 72 °C for 5 minutes. The PCR products (each 10 μl) were sequenced by pyrosequencing using the PSQ24 System (Qiagen) according to manufacturer’s instructions. Methylation status at each of the 4 loci was analyzed individually as a T/C single nucleotide polymorphism (SNP) using QCpG software (Qiagen). Methylation status data at all 4 loci were averaged to provide an overall percentage 5-mC status.

### Intrinsic DNA damage evaluated by neutral comet assay

In order to investigate the possible association of MTHFR variants with intrinsic DNA damage, DNA double-strand breaks were assessed using the comet assay. To this end, mononuclear cells isolated from 10 ml of PBMCs of 13 pSS patients by standard method were analyzed using the single-cell gel electrophoresis assay, performed under neutral conditions as previously described^27^. Briefly, aliquots of 2 × 10^4^ untreated cells were suspended in low melting point agarose (1%) in PBS (135 mM NaCl, 2.5 mM KCl, 10 mM Na_2_HPO4, 1.8 mM KH_2_PO4, pH 7.4) at 37 °C, and spread onto fully frosted microscope slides precoated with a thin layer of 1% normal melting agarose (Biozyme, Hameln, Germany). Then, cells were exposed to neutral lysis buffer (2% sarkosyl, 0.5 M Na_2_EDTA, 0.5 mg/ml proteinase K, pH 8.0) at 37 °C overnight in the dark, electrophoresed under neutral conditions (90 mM Tris buffer, 90 mM boric acid, 2 mM Na_2_EDTA, pH 8.5) for 25 min at 0.6 V/cm, stained with 20μl of 1μg/ml DAPI and analysed with a fluorescence microscope (NIKON Eclipse 400) equipped with a CCD-4230A video camera. Digital images were acquired using an image analysis system (Kinetic Analysis, Wirral, UK). Olive Tail Moments [OTM = (Tail Mean-Head Mean) × (% of DNA)/100] of 100 cells/condition were evaluated.

### Statistical analysis

Genotype frequencies of each MTHFR variant in pSS patients and HC were identified with SNPStats software, using the five genetic models (codominant, dominant, recessive, overdominant and additive)^28^. The allele frequencies in these groups were determined with Shesis software^29^. Odds ratios (OR) and corresponding 95% confidence intervals (CI) were estimated by unconditional logistic regression adjusting for the effects of age and gender. Comparisons of LINE-1 methylation levels and comet assay units according to different MTHFR genotypes were performed by t-tests or Mann Whitney tests, depending on the data distribution, using GraphPad software. Α p-value of less than 0.05 was considered statistically significant.

## Results

### Demographic data

Demographic data are presented in Supplementary Τable S1. As shown, there were no statistically significant differences, regarding age and sex, among groups. Supplementary Τable S2 displays the prevalence of clinical and laboratory features among pSS patients with and without non-Hodgkin’s lymphoma.

### Prevalence of MTHFR polymorphisms in pSS, pSS-lymphoma and HC groups

As shown in Supplementary Τables S3, S4 and S5 similar rates of both MTHFR c. 677C > T and c. 1298A > C polymorphisms among pSS patients, pSS-lymphoma and HC groups were observed. Regarding the allele frequencies of both polymorphisms, no differences among these groups were identified (data not shown). Apart from arthritis, which occurred less frequently in MTHFR TT carriers (36.4 vs 63.6, p: 0.04), no other associations between clinical or serological manifestations and MTHFR variants were observed (data not shown).

### Prevalence of MTHFR polymorphisms in lymphoma subgroups

We next aimed to investigate the potential contribution of the MTHFR variants in distinct lymphoma subgroups’ susceptibility (75 pSS MALT and 19 pSS non-MALT patients). As shown in Table 1, increased frequency of the MTHFR c. 677C > T TT homozygous genotype was detected in the pSS non-MALT group compared to the pSS group [additive model: OR (95% CI): 2.13 (1.06–4.30), p = 0.03], with TT genotype frequencies being 26.3% and 15.7% respectively. No significant differences in the prevalence of the MTHFR c. 677C > T genotype were detected between pSS non-MALT individuals and HC (Table 2), possibly due to the limited number of patients. On the other hand, the MTHFR c. 1298A > C AC and CC genotypes occurred less frequently in the pSS non-MALT patients compared to both pSS patients [additive model: OR (95% CI): 0.31 (0.11–0.88), p = 0.01] (Table 1) and HC [additive model: OR (95% CI): 0.27 (0.09–0.77), p = 0.004] (Table 2).

**Table 1:** Prevalence of MTHFR genotypes in pSS non-MALT and pSS patients, adjusted by gender and age.

SNPs MTHFR	Genotype	HAPMAP Database (%)	pSS non-MALT (n = 19) n (%)	pSS (n=262) n (%)	OR codominant model [95%CI]	p-value	OR dominant model [95%CI]	p-value	OR recessive model [95%CI]	p-value	OR overdominant model [95%CI]	p-value	OR log-additive model [95%CI]	p-value
					**CC vs CT vs TT**		**(CT-TT) vs CC**		**TT vs (CC–CT)**		**CT vs (CC–TT)**			
	CC	46.9	4 (21.1)	102 (38.9)	1.00	0.10	2.70 [0.86-8.43]	0.07	2.74 [0.88-8.51]	0.10	1.29 [0.50-3.31]	0.60	**2.13 [1.06-4.30]**	**0.03**
**c. 677C>T**	CT	44.2	10 (52.6)	119 (45.4)	2.26 [0.68-7.48]									
	TT	8.8	5 (26.3)	41 (15.7)	**4.55 [1.10-18.78]**									
					**AA vs AC vs CC**		**(AC-CC) vs AA**		**CC vs (AA-AC)**		**AC vs (AA-CC)**			
	AA	43.4	15 (79.0)	137 (52.3)	1.00	**0.03**	**0.29 [0.09-0.90]**	**0.02**	0.00 [0.00-NA]	**0.049**	0.44 [0.14-1.37]	0.13	**0.31 [0.11-0.88]**	**0.01**
**c. 1298A>C**	AC	45.1	4 (21.1)	98 (37.4)	0.37 [0.12-1.15]									
	CC	11.5	0 (0.0)	27 (10.3)	0.00 [0.00-NA]									

Genotypes, OR and p-value for the five genetic models (codominant, dominant, recessive, overdominant and additive) were estimated with SNPstats software (statistically significant if p < 0.05).

MTHFR: methylene tetrahydrofolate reductase, pSS: primary Sjogren’s syndrome, MALT: mucosa-associated lymphoid tissue, OR: odds ratio, SNP: single nucleotide polymorphism, VS: versus, HAPMAP: haplotype map.

**Table 2:** Prevalence of MTHFR genotypes in pSS non-MALT and healthy control groups, adjusted by gender and age.

SNPs MTHFR	Genotype	HAPMAP Database (%)	pSS non-MALT (n = 19) n (%)	Healthy controls (n = 600) n (%)	OR codominant model [95%CI]	p-value	OR dominant model [95%CI]	p-value	OR recessive model [95%CI]	p-value	OR overdominant model [95%CI]	p-value	OR log-additive model [95%CI]	p-value
					**CC vs CT vs TT**		**(CT-TT) vs CC**		**TT vs (CC-CT)**		**CT vs (CC-TT)**			
	CC	46.9	4 (21.1)	235 (39.2)	1.00	0.18	2.34 [0.76-7.18]	0.11	2.23 [0.76-6.52]	0.17	1.22 [0.49-3.08]	0.67	1.86 [0.96-3.64]	0.07
**c. 677C>T**	CT	44.2	10 (52.6)	291 (48.5)	2.02 [0.62-6.55]									
	TT	8.8	5 (26.3)	74 (12.3)	3.50 [0.89-13.65]									
					**AA vs AC vs CC**		**(AC-CC) vs AA**		**CC vs (AA-AC)**		**AC vs (AA-CC)**			
	AA	43.4	15 (79.0)	273 (45.5)	1.00	**0.01**	**0.25 [0.08-0.78]**	**0.008**	0.00 [0.00-NA]	0.06	0.37 [0.12-1.14]	0.06	**0.27 [0.09-0.77]**	**0.004**
**c. 1298A>C**	AC	45.1	4 (21.1)	266 (44.3)	**0.31 [0.10-0.95]**									
	CC	11.5	0 (0.0)	61 (10.2)	0.00 [0.00-NA]									

Genotypes, OR and p-value for the five genetic models (codominant, dominant, recessive, overdominant and additive) were estimated with SNPstats software (statistically significant if p < 0.05).

MTHFR: methylene tetrahydrofolate reductase, pSS: primary Sjogren’s syndrome, MALT: mucosa-associated lymphoid tissue, OR: odds ratio, SNP: single nucleotide polymorphism, vs: versus, HAPMAP: haplotype map.

Table 3 displays the results of allele analysis of the studied MTHFR variants, comparing the pSS non-MALT group with HC as well as with pSS patients, respectively. The pSS non-MALT group exhibited increased MTHFR c. 677C > T T allele prevalence compared to HC [OR (95% CI): 1.93 (1.01–3.68), p = 0.04], with the allele frequencies being 52.6% and 36.6%, respectively. On the other hand, reduced rates of the minor MTHFR c. 1298A > C C allele were revealed in the pSS non-MALT group compared to both HC group [OR (95% CI): 0.25 (0.09–0.70), p = 0.004] and pSS patients [OR (95% CI): 0.29 (0.10–0.83), p = 0.01], suggesting a protective role against non-MALT development. The corresponding allele frequencies in pSS non-MALT, pSS and HC were 10.5%, 29% and 32.3% respectively.

**Table 3:** Prevalence of MTHFR c. 677C > T T and MTHFR c. 1298A > C C allele in the pSS non-MALT patients, pSS patients and healthy control group.

SNPs MTHFR	Allele	HAPMAP Database (%)	pSS non-MALT (n = 19) (%)	pSS (n = 262) (%)	Healthy controls (n = 600) (%)	OR 1 [95% CI]	p-value 1	OR 2 [95% CI]	p-value 2
c. 677C > T	C	69.0	47.4	61.6	63.4	1.79 [0.92–3.46]	0.08	**1.93 [1.01–3.68]**	**0.04**
	T	31.0	52.6	38.4	36.6				
c. 1298A > C	A	65.9	89.5	71.0	67.7	**0.29 [0.10–0.83]**	**0.01**	**0.25 [0.09–0.70]**	**0.004**
	C	34.1	10.5	29.0	32.3				

Allele analysis was performed with Shesis software (statistically significant if p < 0.05).

OR 1, p-value 1: pSS non-MALT vs pSS, OR 2, p-value 2: pSS non-MALT vs Healthy Controls.

MTHFR: methylene tetrahydrofolate reductase, pSS: primary Sjogren’s syndrome. MALT: mucosa-associated lymphoid tissue, SNP: single nucleotide polymorphisms, OR: odds ratio, HAPMAP: haplotype map, vs: versus.

No differences in the prevalence of MTHFR c. 677C > T and c. 1298A > C polymorphisms at both genotype and allele level were observed when the pSS MALT group was compared with pSS patients and HC (Supplementary Τables S6 and S7).

When MALT and nodal MZL were combined together and compared to other types of lymphoma, no statistically significant differences in MTHFR variant frequencies were observed. Similarly, no statistically significant differences were detected between DLBCL cases, SS and HC (Supplementary Τables S8–S13).

### MTHFR 677TT and 1298AC variants: Associations with global DNA methylation status and DNA damage

**Figure 1:**
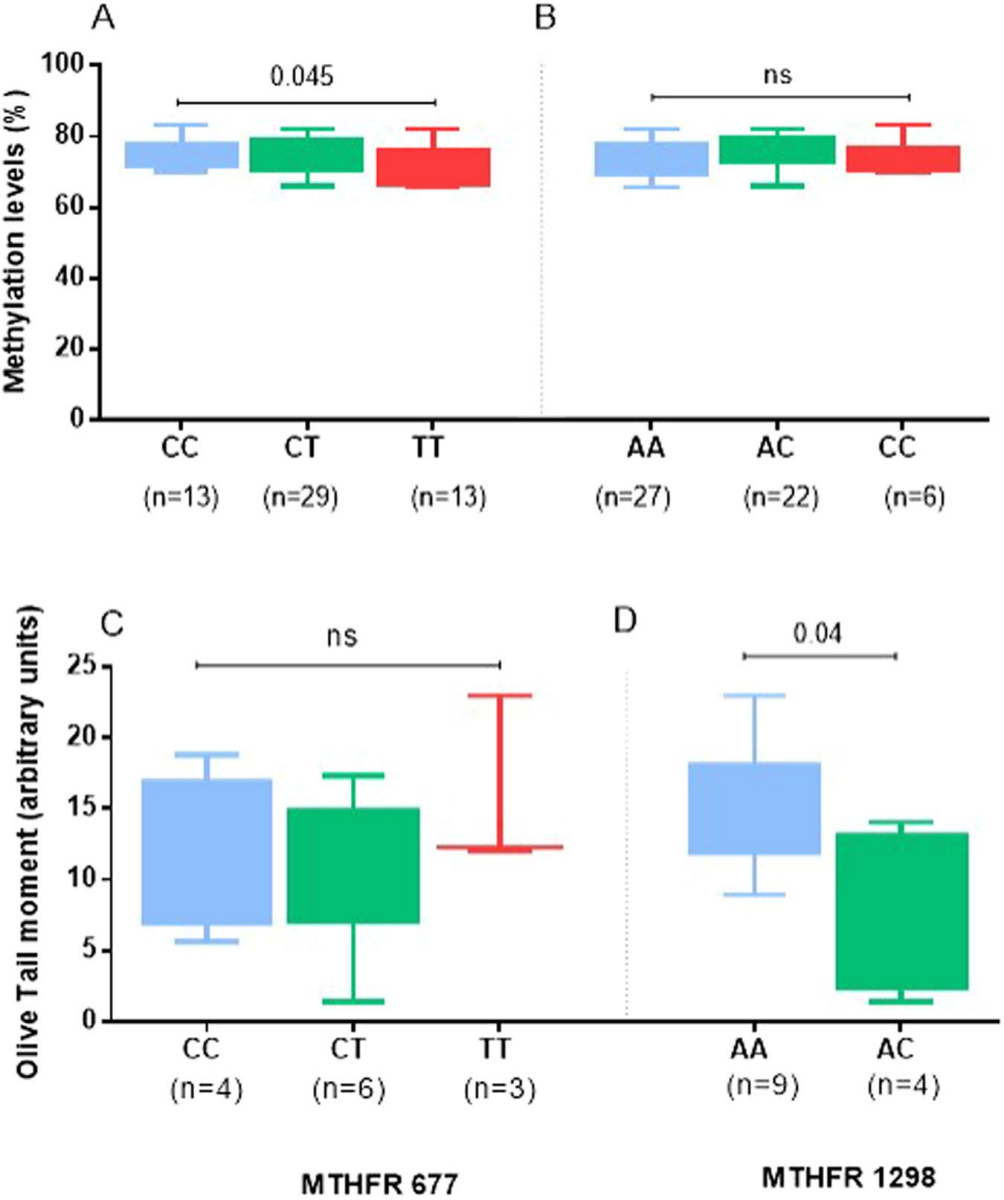
Long interspersed nuclear element 1 (LINE-1) promoter methylation according to MTHFR genotypes and intrinsic DNA damage levels in pSS patients according to MTHFR genotypes. Panel A: Significantly lower DNA methylation levels among individuals bearing the MTHFR c. 677C > T TT genotype, compared to the CC carriers were observed (TT vs CC: 71.57 ± 5.43 vs 75.56 ± 4.13, p = 0.045). Panel B: No statistically significant differences in DNA methylation levels of LINE-1 promoter were observed among c. 1298A > C CC, AC and AA genotype groups (CC vs AC vs AA: 73.70 ± 5.00 vs 75.42 ± 4.89 vs 73.41 ± 4.98, all comparisons non significant). Panel C: No statistically significant differences in DNA double-strand break levels (expressed as Olive Tail Moments, arbitrary units) between MTHFR c. 677C > T genotypes (CC vs CT vs TT: 11.67 ± 5.40 vs 11.23 ± 5.53 vs 15.74 ± 6.30). Panel D: Decreased levels of comet assay units among pSS patients carrying the MTHFR c. 1298A > C AC genotype compared to those bearing the c. 1298A > C AA genotype (AA vs AC: 14.41 ± 4.38 vs 7.89 ± 5.54, p = 0.04). MTHFR: methylene tetrahydrofolate reductase, pSS: primary Sjogren’s syndrome.

Given that MTHFR 677TT and 1298CC/AC genotypes -previously related to altered genomic methylation levels^23^- were found to be associated with pSS non-MALT lymphoma, we next decided to explore the functional implications of these variants. To this end, we measured LINE-1 promoter methylation status (as a marker of global methylation) in genomic DNA derived from MSG tissues from pSS patients with different MTHFR c. 677C > T and c. 1298A > C genotypes. MSG biopsies were obtained at the time of pSS diagnosis. In some of them the diagnosis of *in situ* MALT lymphoma was concomitant to the pSS diagnosis. Focus score (mean ± SD) was 1.995 ± 1.351 in the pSS group and 4.473 ± 2.411 in the pSS lymphoma group (p < 0.001). Nevertheless, the mean methylation levels were not significantly different between pSS and pSS lymphoma groups (74.2 ± 4.8 vs 74.3 ± 5.1, p-value: 0.96). As shown in Fig. 1A, the mean methylation levels were significantly lower in individuals bearing the MTHFR c. 677C > T TT homozygous genotype compared to those with the c. 677C > T CC genotype (TT vs CC: 71.57 ± 5.43 vs 75.56 ± 4.13, p = 0.045). Regarding the MTHFR c. 1298A > C polymorphism, no statistically significant differences in methylation levels of LINE-1 promoter were observed in the three genotype groups (CC vs AC vs AA: 73.70 ± 5.00 vs 75.42 ± 4.89 vs 73.41 ± 4.98, all comparisons non significant, Fig. 1B).

Additionally, we sought to investigate whether MTHFR 677TT and 1298CC/AC genotypes could be linked to increased DNA damage levels, providing a plausible biological scenario for the observed association with non-MALT lymphomagenesis in the setting of pSS. Towards this direction, DNA double-strand break levels were quantified in PBMCs derived from 13 pSS patients by comet assay. As displayed in Fig. 1D, DNA double-strand breaks levels (expressed as Olive Tail Moments, arbitrary units) were found to be significantly decreased in individuals carrying the MTHFR c. 1298A > C AC genotype (AA vs AC: 14.41 ± 4.38 vs 7.89 ± 5.54, p = 0.04), again suggesting that this particular MTHFR variant may have a protective role by restricting double-strand break formation. No significant differences were detected between distinct MTHFR c. 677C > T genotypes (CC vs CT vs TT: 11.67 ± 5.40 vs 11.23 ± 5.53 vs 15.74 ± 6.30, Fig. 1C).

## Discussion

To our knowledge, the current study is the first to explore the potential role of MTHFR gene variants in both pSS and pSS-related lymphomagenesis. Whereas no differences in the prevalence of MTHFR c. 677C > T and c. 1298A > C polymorphisms were detected among pSS, pSS-lymphoma patients (as a whole) and HC, further analysis, according to the lymphoma subtype, revealed increased frequency of the MTHFR c. 677C > T TT genotype and T allele in the pSS non-MALT group compared to pSS patients and HC respectively. Furthermore, reduced prevalence of the MTHFR c. 1298A > C AC and CC genotypes and C allele in the pSS non-MALT group compared to pSS and HC groups was identified, with no differences being detected in the pSS MALT group compared to the other groups. Therefore, the reduced global DNA methylation status in the MTHFR677TT genetic variant and possibly the lack of the protective role of MTHFR1298AC in decreasing DNA double-strand break levels in those pSS patients who develop non-MALT, suggest a role for MTHFR variants in non-MALT lymphomagenesis in the setting of pSS.

In line with our findings, several studies -mainly in Caucasians- reported that the c. 677C > T TT genotype or T allele conferred increased risk for non-MALT NHL subtypes^18–21^, whereas others, carried out in Asian populations, demonstrated a protective role of the c. 677C > T genetic variant in DLBCL^30, 31^ and FL^32^ development. However, other studies did not reach similar conclusions neither in Caucasian^33–35^, nor in Asian populations^36, 37^. In accord with our observations, Skibola *et al*.^18^ also reported reduced frequencies of MTHFR c. 1298A > C variants in NHL Caucasian populations, opposite to the findings in individuals of Asian descent^36, 37^. In a recent metaanalysis^38^, after stratification by ethnicity, MTHFR c. 677C > T polymorphism conferred increased risk for NHL development in Caucasians, while MTHFR c. 1298A > C polymorphism increased NHL susceptibility in Asian populations, indicating that MTHFR related risk for lymphoma development is race dependent.

No statistically significant associations between MTHFR genotypes and disease characteristics were observed. This lack of association was not however surprising since classical predictors for lymphoma development, such as purpura, low C4 and cryoglobulinemia have been previously shown to be associated only with Marginal zone lymphomas^13^.

Also, we aimed to explore whether changes in DNA damage levels could be related to the altered frequencies of the MTHFR variants. Indeed, we found that mononuclear cells from MTHFR c. 1298A > C AC pSS carriers are characterized by decreased DNA double-strand breaks levels. Since DNA double-strand breaks promote chromosomal instability, translocations, and aberrations that may contribute to increased cancer risk^39^, these data are in line with the reduced frequency of the MTHFR c. 1298A > C AC genotype in pSS non-MALT patients observed in the present study. On the other hand, in accordance with previous data showing no influence of the MTHFR c. 677C > T polymorphism on chromosome damage^40^ and DNA uracil incorporation^41^, we found no significant differences in the DNA double-strand break levels between distinct MTHFR c. 677C > T genotypes.

DNA methylation is considered a major epigenetic mechanism, alterations of which result in increased transcription or silencing of specific genes. Aberrant DNA methylation, and mainly hypomethylation, has been implicated in the pathogenesis of systemic autoimmune diseases^14, 15^, including SS^42^, lupus^43^ and RA^44^. In pSS, hypomethylation of the TNFSF7 gene, as well as of interferon type I inducible genes in CD4^+^ T lymphocytes, have been proposed as potential pathogenic contributors^45, 46^. Likewise, DNA methylation modifications, leading either to overexpression of oncogenes and/or defective expression of tumor suppressor genes, constitute an important pathogenic underlying mechanism of lymphoproliferative disorders^16^.

Given that MTHFR c. 677C > T TT genotypes^47^ through decreasing enzyme activity, are considered to promote DNA hypomethylation, we next evaluated the mean methylation levels of the LINE-1 promoter, a surrogate marker of global DNA methylation levels^25^. Indeed, our findings denoted a significant association of the c. 677C > T TT genotype with DNA hypomethylation implying that increased frequencies of MTHFR c. 677C > T polymorphisms in the pSS-NHL non MALT could lead to reduced global methylation levels. Previous studies on DLBCL patients of Italian and Saudi descent, failed to demonstrate an effect of MTHFR polymorphisms in DNA methylation levels of a particular tumor suppressor gene, the O-6-methylguanine-DNA methyltranferase (MGMT)^34, 36^. An heterogeneous effect of MTHFR c. 677C > T polymorphism in DNA methylation patterns was observed in studies conducted in solid tumors patients: T allele was associated with hypomethylation of several oncogenes, such as C-erb-2, Insulin-like growth factor 2 (IGF-2) and B-cell lymphoma 2 (Bcl-2)^48–50^, and TT genotype was found to enhance methylation of tumor suppressor genes, such as MGMT^51, 52^.

Unfortunately, because of the limited available number of pSS non-MALT patients due to the rarity of this complication, we were not able to directly detect associations of the MTHFR variants with methylation and DNA damage in this subgroup. Furthermore, since MTHFR polymorphisms may affect methylation status through interaction with folate levels^53^, information on folate intake and folate concentrations would be desirable.

In conclusion, the current study presents novel associations of MTHFR polymorphisms with non-MALT lymphoma in the setting of pSS. MTHFR-mediated DNA hypomethylation and genetic stability pathway defects could contribute to pSS-related non-MALT lymphomagenesis, revealing that the pathogenic nature of pSS-related lymphoma subtypes is distinct.

## Electronic supplementary material

Supplementary tables
