# Supplementary material for: MTHFR gene variants and non-MALT lymphoma development in primary Sjogren’s syndrome
Source: Sci Rep. 2017 Aug 4;7:7354. doi: 10.1038/s41598-017-07347-w (PMC5544668; doi:10.1038/s41598-017-07347-w)
Supplement: Supplementary file 1 — Supplementary tables [file 41598_2017_7347_MOESM1_ESM.doc]

**MTHFR gene variants and non-MALT lymphoma development in primary Sjogren’s syndrome**

Sofia Fragkioudaki^1^, Adrianos Nezos^1^, Vassilis L. [Souliotis](http://www.ncbi.nlm.nih.gov/pubmed/?term=Souliotis VL%5BAuthor%5D&cauthor=true&cauthor_uid=27492607)^2^, Ilenia Chatziandreou^3^ Aggeliki A. Saetta^3^, Nikolaos Drakoulis^4^, Athanasios G.Tzioufas^5,6^, Michael Voulgarelis^5,6^, Petros P. Sfikakis^6,7^, Michael Koutsilieris^1^, Mary K. Crow^8^, Haralampos M.Moutsopoulos^5^, ^*^Clio P. Mavragani^1,5,6^

^1^Department of Physiology, School of Medicine, National and Kapodistrian University of Athens, Athens, Greece.

^2^Institute of Biology, Medicinal Chemistry and Biotechnology, National Hellenic Research Foundation, Athens, Greece.

^3^Department of Pathology, School of Medicine, National and Kapodistrian University of Athens, Athens, Greece.

^4^Department of Pharmaceutical Technology, Faculty of Pharmacy, National and Kapodistrian University of Athens, Athens, Greece.

^5^Department of Pathophysiology, School of Medicine, National and Kapodistrian University of Athens, Athens, Greece.

^6^Joint Academic Rheumatology Program, National and Kapodistrian University of Athens School of Medicine, Athens, Greece.

^7^First Department of Propaedeutic Internal Medicine, National and Kapodistrian University of Athens School of Medicine, Athens, Greece.

^8^Mary Kirkland Center for Lupus Research, Hospital for Special Surgery, Weill Medical College of Cornell University, New York, NY, USA.

**Corresponding author-Reprint Requests**

Clio P. Mavragani, MD, Department of Physiology, School of Medicine, University of Athens, M.Asias 75, 11527, Athens, Greece, Tel: +30-210-746 2714, FAX: +30-210-746 2571, e-mail: kmauragan@med.uoa.gr

**Supplementary Table S1.** Demographic data of pSS patients and healthy control group.

|  | **pSS (n=262)** | **pSS-lymphoma (n=94)** | | **Healthy controls (n=600)** | **p-value** | | | | |
| --- | --- | --- | --- | --- | --- | --- | --- | --- | --- |
|  |  | **pSS MALT (n=75)** | **pSS non-MALT (n=19)** |  | **p1** | **p2** | **p3** | **p4** | **p5** |
| Age (mean±SD) | 62.4±13.7 | 61.2±12.4 | 68.1±12.3 | 63.5±10.4 | ns | ns | ns | ns | ns |
| Females/Males (F:M ratio) | 243/19 (~13:1) | 70/5 (~14:1) | 19/0 | 536/64 (~8:1) | ns | ns | ns | ns | ns |

p1: pSS vs HC, p2: pSS MALT vs HC, p3: pSS non-MALT vs HC, p4: pSS vs pSS MALT, p5: pSS vs pSS non-MALT

pSS: primary Sjogren’s syndrome, MALT: mucosa-associated lymphoid tissue, SD: standard deviation, ns: non significant

**Supplementary Table S2.** Prevalence of clinical and laboratory features in pSS patients with and without non-Hodgkin’s lymphoma.

| **Clinical and laboratory features** | **pSS**  **(n=262)** | **pSS NHL**  **(n=94)** | **OR [95%CI]** | **p-value** |
| --- | --- | --- | --- | --- |
| **Age (mean±SD)** | 62.39±13.7 | 62.6±12.6 |  | .70 |
| **Disease duration (years±SD)** | 10.7±6.7 | 12.6±7.6 |  | .04 |
| **Oral dryness n (%)** | 239 (91.2) | 91 (96.8) | 2.92 [0.86-9.96] | .10 |
| **Ocular dryness n (%)** | 234 (89.3) | 87 (92.6) | 1.49 [0.63-3.53] | .43 |
| **SGE n (%)** | 58 (22.1) | 61 (64.9) | 6.50 [3.89-10.87] | <.001 |
| **Palpable purpura n (%)** | 31 (11.8) | 38 (40.4) | 5.06 [2.89-8.83] | <.001 |
| **Anti-Ro positivity n (%)** | 178/254 (70.1) | 84/92 (91.3) | 4.48 [2.07-9.71] | <.001 |
| **Anti-La positivity n (%)** | 93/254 (36.6) | 48/92 (52.2) | 1.89 [1.17-3.06] | 0.01 |
| **Cryoglobulinemia n (%)** | 12/200 (6.0) | 27/83 (32.5) | 7.55 [3.59-15.87] | <.001 |
| **Monoclonal Gammopathy n (%)** | 16/239 (6.7) | 21/91 (23.1) | 4.18 [2.07-8.45] | <.001 |
| **RF positivity n (%)** | 121/233 (51.9) | 77/90 (85.6) | 5.48 [2.89-10.4] | <.001 |
| **C4 hypocomplementemia n (%)** | 129/250 (51.6) | 72/90 (80.0) | 3.75 [2.12-6.65] | <.001 |
| **MSG biopsy Focus score (mean±SD)** | 3.07±3.2 | 2.01±1.9 |  | .08 |
| **MSG biopsy Tarpley score (mean±SD)** | 1.9±1.0 | 2.8±0.9 |  | <.001 |

pSS: Primary Sjogren’s syndrome, NHL: non-Hodgkin’s lymphoma, SGE: salivary gland enlargement, RF: rheumatoid factor, MSG: minor salivary gland, SD: standard deviation, OR: odds ratio, CI: confidence interval

**Supplementary Table S3.** Prevalence of MTHFR genotypes in pSS patients and healthy controls, adjusted by gender and age. Genotypes, ORs and p-values for the five genetic models (codominant, dominant, recessive, overdominant and additive) were estimated with SNPstats software (statistically significant if p<0.05).

| **SNPs MTHFR** | **Genotype** | **HAPMAP Database (%)** | **pSS**  **(n=262) n (%)** | **Healthy controls (n=600) n (%)** | **OR codominant model [95%CI]** | **p-value** | **OR dominant model [95%CI]** | **p-value** | **OR recessive model [95%CI]** | **p-value** | **OR overdominant model [95%CI]** | **p-value** | **OR log-additive model [95%CI]** | **p-value** |
| --- | --- | --- | --- | --- | --- | --- | --- | --- | --- | --- | --- | --- | --- | --- |
|  |  |  |  |  | **CC vs CT vs TT** |  | **(CT-TT) vs CC** |  | **TT vs (CC-CT)** |  | **CT vs (CC-TT)** |  |  |  |
|  | CC | 46.9 | 102 (38.9) | 235 (39.2) | 1.00 | 0.42 | 1.03 [0.77-1.40] | 0.82 | 1.32 [0.87-2.00] | 0.19 | 0.90 [0.67-1.21] | 0.49 | 1.09 [0.88-1.36] | 0.41 |
| **c. 677C>T** | CT | 44.2 | 119 (45.4) | 291 (48.5) | 0.97 [0.70-1.33] |
|  | TT | 8.8 | 41 (15.7) | 74 (12.3) | 1.30 [0.83-2.03] |
|  |  |  |  |  | **AA vs AC vs CC** |  | **(AC-CC) vs AA** |  | **CC vs (AA-AC)** |  | **AC vs (AA-CC)** |  |  |  |
|  | AA | 43.4 | 137 (52.3) | 273 (45.5) | 1.00 | 0.17 | 0.76 [0.57-1.02] | 0.07 | 0.96 [0.60-1.56] | 0.88 | 0.76 [0.57-1.03] | 0.08 | 0.85 [0.68-1.06] | 0.15 |
| **c. 1298A>C** | AC | 45.1 | 98 (37.4) | 266 (44.3) | 0.74 [0.54-1.01] |
|  | CC | 11.5 | 27 (10.3) | 61 (10.2) | 0.84 [0.51-1.39] |

MTHFR: methylene tetrahydrofolate reductase, pSS: primary Sjogren’s syndrome, OR: odds ratio, SNP: single nucleotide polymorphism, VS: versus, HAPMAP: haplotype map

**Supplementary Table S4.** Similar MTHFR genotype frequencies between pSS-lymphoma patients and healthy controls, adjusted by gender and age. Genotypes, ORs and p-values for the five genetic models (codominant, dominant, recessive, overdominant and additive) were estimated with SNPstats software (statistically significant if p<0.05).

| **SNPs MTHFR** | **Genotypes** | **HAPMAP Database (%)** | **pSS lymphoma (n=94) n (%)** | **Healthy controls (n=600) n (%)** | **OR codominant model [95%CI]** | **p-value** | **OR dominant model [95%CI]** | **p-value** | **OR recessive model [95%CI]** | **p-value** | **OR overdominant model [95%CI]** | **p-value** | **OR log-additive model [95%CI]** | **p-value** |
| --- | --- | --- | --- | --- | --- | --- | --- | --- | --- | --- | --- | --- | --- | --- |
|  |  |  |  |  | **CC vs CT vs TT** |  | **(CT-TT) vs CC** |  | **TT vs (CC-CT)** |  | **CT vs (CC-TT)** |  |  |  |
|  | CC | 46.9 | 36 (38.3) | 235 (39.2) | 1.00 | 0.75 | 1.07 [0.68-1.67] | 0.78 | 1.28 [0.68-2.38] | 0.45 | 0.95 [0.61-1.47] | 0.81 | 1.10 [0.80-1.52] | 0.56 |
| **c. 677C>T** | CT | 44.2 | 44 (46.8) | 291 (48.5) | 1.01 [0.63-1.63] |
|  | TT | 8.8 | 14 (14.9) | 74 (12.3) | 1.28 [0.65-2.52] |
|  |  |  |  |  | **AA vs AC vs CC** |  | **(AC-CC) vs AA** |  | **CC vs (AA-AC)** |  | **AC vs (AA-CC)** |  |  |  |
|  | AA | 43.4 | 49 (52.1) | 273 (45.5) | 1.00 | 0.50 | 0.77 [0.50-1.19] | 0.24 | 0.89 [0.43-1.87] | 0.76 | 0.80 [0.51-1.25] | 0.32 | 0.84 [0.60-1.18] | 0.30 |
| **c. 1298A>C** | AC | 45.1 | 36 (38.3) | 266 (44.3) | 0.77 [0.48-1.22] |
|  | CC | 11.5 | 9 (9.6) | 61 (10.2) | 0.79 [0.37-1.70] |

MTHFR: methylene tetrahydrofolate reductase, pSS: primary Sjogren’s syndrome, OR: odds ratio, SNP: single nucleotide polymorphism, VS: versus, HAPMAP: haplotype map

**Supplementary Table S5.** Similar MTHFR genotype prevalence in pSS-lymphoma and pSS patients, adjusted by gender and age. Genotypes, ORs and p-values for the five genetic models (codominant, dominant, recessive, overdominant and additive) were estimated with SNPstats software (statistically significant if p<0.05).

| **SNPs MTHFR** | **Genotype** | **HAPMAP Database (%)** | **pSS lymphoma (n=94) n (%)** | **pSS**  **(n=262) n (%)** | **OR codominant model [95%CI]** | **p-value** | **OR dominant model [95%CI]** | **p-value** | **OR recessive model [95%CI]** | **p-value** | **OR overdominant model [95%CI]** | **p-value** | **OR log-additive model [95%CI]** | **p-value** |
| --- | --- | --- | --- | --- | --- | --- | --- | --- | --- | --- | --- | --- | --- | --- |
|  |  |  |  |  | **CC vs CT vs TT** |  | **(CT-TT) vs CC** |  | **TT vs (CC-CT)** |  | **CT vs (CC-TT)** |  |  |  |
|  | CC | 46.9 | 36 (38.3) | 102 (38.9) | 1.00 | 0.96 | 1.04 [0.64-1.70] | 0.86 | 0.95 [0.49-1.86] | 0.89 | 1.07 [0.66-1.72] | 0.79 | 1.01 [0.72-1.42] | 0.96 |
| **c. 677C>T** | CT | 44.2 | 44 (46.8) | 119 (45.4) | 1.06 [0.64-1.78] |
|  | TT | 8.8 | 14 (14.9) | 41 (15.7) | 0.99 [0.48-2.04] |
|  |  |  |  |  | **AA vs AC vs CC** |  | **(AC-CC) vs AA** |  | **CC vs (AA-AC)** |  | **AC vs (AA-CC)** |  |  |  |
|  | AA | 43.4 | 49 (52.1) | 137 (52.3) | 1.00 | 0.96 | 1.01 [0.63-1.62] | 0.97 | 0.90 [0.41-2.00] | 0.80 | 1.05 [0.64-1.71] | 0.85 | 0.98 [0.69-1.40] | 0.93 |
| **c. 1298A>C** | AC | 45.1 | 36 (38.3) | 98 (37.4) | 1.03 [0.63-1.71] |
|  | CC | 11.5 | 9 (9.6) | 27 (10.3) | 0.91 [0.40-2.09] |

MTHFR: methylene tetrahydrofolate reductase, pSS: primary Sjogren’s syndrome, OR: odds ratio, SNP: single nucleotide polymorphism, VS: versus, HAPMAP: haplotype map

**Supplementary Table S6.** Similar MTHFR genotype frequencies in pSS MALT patients and healthy controls group, adjusted by gender and age. Genotypes, ORs and p-values for the five genetic models (codominant, dominant, recessive, overdominant and additive) were estimated with SNPstats software (statistically significant if p<0.05).

| **SNPs MTHFR** | **Genotype** | **HAPMAP Database (%)** | **pSS MALT (n=75) n (%)** | **Healthy controls (n=600) n (%)** | **OR codominant model [95%CI]** | **p-value** | **OR dominant model [95%CI]** | **p-value** | **OR recessive model [95%CI]** | **p-value** | **OR overdominant model [95%CI]** | **p-value** | **OR log-additive model [95%CI]** | **p-value** |
| --- | --- | --- | --- | --- | --- | --- | --- | --- | --- | --- | --- | --- | --- | --- |
|  |  |  |  |  | **CC vs CT vs TT** |  | **(CT-TT) vs CC** |  | **TT vs (CC-CT)** |  | **CT vs (CC-TT)** |  |  |  |
|  | CC | 46.9 | 32 (42.7) | 235 (39.2) | 1.00 | 0.89 | 0.90 [0.55-1.46] | 0.67 | 1.04 [0.49-2.18] | 0.93 | 0.89 [0.55-1.44] | 0.63 | 0.95 [0.66-1.37] | 0.78 |
| **c. 677C>T** | CT | 44.2 | 34 (45.3) | 291 (48.5) | 0.88 [0.53-1.48] |
|  | TT | 8.8 | 9 (12.0) | 74 (12.3) | 0.97 [0.44-2.13] |
|  |  |  |  |  | **AA vs AC vs CC** |  | **(AC-CC) vs AA** |  | **CC vs (AA-AC)** |  | **AC vs (AA-CC)** |  |  |  |
|  | AA | 43.4 | 34 (45.3) | 273 (45.5) | 1.00 | 0.94 | 0.99 [0.61-1.60] | 0.96 | 1.12 [0.53-2.38] | 0.76 | 0.94 [0.58-1.53] | 0.81 | 1.02 [0.71-1.46] | 0.91 |
| **c. 1298A>C** | AC | 45.1 | 32 (42.7) | 266 (44.3) | 0.96 [0.57-1.61] |
|  | CC | 11.5 | 9 (12.0) | 61 (10.2) | 1.10 [0.50-2.43] |

MTHFR: methylene tetrahydrofolate reductase, pSS: primary Sjogren’s syndrome, MALT: mucosa-associated lymphoid tissue, OR: odds ratio, SNP: single nucleotide polymorphism, VS: versus, HAPMAP: haplotype map

**Supplementary Table S7.** Similar MTHFR genotype frequencies in pSS MALT and pSS patients, adjusted by gender and age. Genotypes, ORs and p-values for the five genetic models (codominant, dominant, recessive, overdominant and additive) were estimated with SNPstats software (statistically significant if p<0.05).

| **SNPs MTHFR** | **Genotype** | **HAPMAP Database (%)** | **pSS MALT (n=75) n (%)** | **pSS (n=262) n (%)** | **OR codominant model [95%CI]** | **p-value** | **OR dominant model [95%CI]** | **p-value** | **OR recessive model [95%CI]** | **p-value** | **OR overdominant model [95%CI]** | **p-value** | **OR log-additive model [95%CI]** | **p-value** |
| --- | --- | --- | --- | --- | --- | --- | --- | --- | --- | --- | --- | --- | --- | --- |
|  |  |  |  |  | **CC vs CT vs TT** |  | **(CT-TT) vs CC** |  | **TT vs (CC-CT)** |  | **CT vs (CC-TT)** |  |  |  |
|  | CC | 46.9 | 32 (42.7) | 102 (38.9) | 1.00 | 0.62 | 0.85 [0.50-1.43] | 0.54 | 0.70 [0.32-1.53] | 0.35 | 1.01 [0.60-1.70] | 0.97 | 0.84 [0.58-1.23] | 0.37 |
| **c. 677C>T** | CT | 44.2 | 34 (45.3) | 119 (45.4) | 0.91 [0.52-1.59] |
|  | TT | 8.8 | 9 (12.0) | 41 (15.7) | 0.66 [0.29-1.53] |
|  |  |  |  |  | **AA vs AC vs CC** |  | **(AC-CC) vs AA** |  | **CC vs (AA-AC)** |  | **AC vs (AA-CC)** |  |  |  |
|  | AA | 43.4 | 34 (45.3) | 137 (52.3) | 1.00 | 0.55 | 1.34 [0.80-2.24] | 0.27 | 1.16 [0.52-2.60] | 0.72 | 1.28 [0.75-2.16] | 0.37 | 1.20 [0.83-1.75] | 0.33 |
| **c. 1298A>C** | AC | 45.1 | 32 (42.7) | 98 (37.4) | 1.34 [0.77-2.33] |
|  | CC | 11.5 | 9 (12.0) | 27 (10.3) | 1.32 [0.56-3.07] |

MTHFR: methylene tetrahydrofolate reductase, pSS: primary Sjogren’s syndrome, MALT: mucosa-associated lymphoid tissue, OR: odds ratio, SNP: single nucleotide polymorphism, VS: versus, HAPMAP: haplotype map

**Supplementary Table S8.** MTHFR prevalence in pSS MALT+ nMZL patients compared to pSS patients. Genotypes, ORs and p-values for the five genetic models (codominant, dominant, recessive, overdominant and additive) were estimated with SNPstats software (statistically significant if p<0.05).

| **SNPs MTHFR** | **Genotype** | **HAPMAP Database (%)** | **pSS MALT+ nMZL (n=79) n (%)** | **pSS (n=262) n (%)** | **OR codominant model [95%CI]** | **p-value** | **OR dominant model [95%CI]** | **p-value** | **OR recessive model [95%CI]** | **p-value** | **OR overdominant model [95%CI]** | **p-value** | **OR log-additive model [95%CI]** | **p-value** |
| --- | --- | --- | --- | --- | --- | --- | --- | --- | --- | --- | --- | --- | --- | --- |
|  |  |  |  |  | **CC vs CT vs TT** |  | **(CT-TT) vs CC** |  | **TT vs (CC-CT)** |  | **CT vs (CC-TT)** |  |  |  |
|  | CC | 46.9 | 32 (40.5) | 102 (38.9) | 1.00 | 0.75 | 0.93 [0.56-1.57] | 0.80 | 0.75 [0.35-1.59] | 0.45 | 1.08  [0.65-1.78] | 0.78 | 0.90  [0.62-1.30] | 0.57 |
| **c. 677C>T** | CT | 44.2 | 37 (46.8) | 119 (45.4) | 1.00 [0.58-1.72] |
|  | TT | 8.8 | 10 (12.7) | 41 (15.7) | 0.75 [0.33-1.68] |
|  |  |  |  |  | **AA vs AC vs CC** |  | **(AC-CC) vs AA** |  | **CC vs (AA-AC)** |  | **AC vs (AA-CC)** |  |  |  |
|  | AA | 43.4 | 38 (48.1) | 137 (52.3) | 1.00 | 0.79 | 1.19 [0.72-1.97] | 0.50 | 1.09 [0.49-2.44] | 0.83 | 1.16  [0.69-1.95] | 0.57 | 1.12  [0.77-1.62] | 0.55 |
| **c. 1298A>C** | AC | 45.1 | 32 (40.5) | 98 (37.4) | 1.20 [0.70-2.05] |
|  | CC | 11.5 | 9 (11.4) | 27 (10.3) | 1.18 [0.51-2.72] |

MTHFR: methylene tetrahydrofolate reductase, pSS: primary Sjogren’s syndrome, MALT: mucosa-associated lymphoid tissue, nMZL: nodal marginal zone lymphoma, OR: odds ratio, SNP: single nucleotide polymorphism, VS: versus, HAPMAP: haplotype map

**Supplementary Table S9.** MTHFR prevalence pSS MALT+ nMZL patients compared to healthy controls. Genotypes, OR and p-value for the five genetic models (codominant, dominant, recessive, overdominant and additive) were estimated with SNPstats software (statistically significant if p<0.05).

| **SNPs MTHFR** | **Genotype** | **HAPMAP Database (%)** | **pSS MALT+ nMZL (n=79) n (%)** | **Healthy controls (n=600) n (%)** | **OR codominant model [95%CI]** | **p-value** | **OR dominant model [95%CI]** | **p-value** | **OR recessive model [95%CI]** | **p-value** | **OR overdominant model [95%CI]** | **p-value** | **OR log-additive model [95%CI]** | **p-value** |
| --- | --- | --- | --- | --- | --- | --- | --- | --- | --- | --- | --- | --- | --- | --- |
|  |  |  |  |  | **CC vs CT vs TT** |  | **(CT-TT) vs CC** |  | **TT vs (CC-CT)** |  | **CT vs (CC-TT)** |  |  |  |
|  | CC | 46.9 | 32 (40.5) | 235 (39.2) | 1.00 | 0.96 | 0.98 [0.61-1.59] | 0.94 | 1.09 [0.54-2.22] | 0.81 | 0.95 [0.59-1.52] | 0.82 | 1.01 [0.71-1.44] | 0.95 |
| **c. 677C>T** | CT | 44.2 | 37 (46.8) | 291 (48.5) | 0.96 [0.58-1.59] |
|  | TT | 8.8 | 10 (12.7) | 74 (12.3) | 1.07 [0.50-2.29] |
|  |  |  |  |  | **AA vs AC vs CC** |  | **(AC-CC) vs AA** |  | **CC vs (AA-AC)** |  | **AC vs (AA-CC)** |  |  |  |
|  | AA | 43.4 | 38 (48.1) | 273 (45.5) | 1.00 | 0.84 | 0.89 [0.55-1.42] | 0.62 | 1.06 [0.50-2.24] | 0.87 | 0.86 [0.54-1.40] | 0.55 | 0.95 [0.66-1.35] | 0.77 |
| **c. 1298A>C** | AC | 45.1 | 32 (40.5) | 266 (44.3) | 0.86 [0.52-1.42] |
|  | CC | 11.5 | 9 (11.4) | 61 (10.2) | 0.99 [0.45-2.17] |

MTHFR: methylene tetrahydrofolate reductase, pSS: primary Sjogren’s syndrome, MALT: mucosa-associated lymphoid tissue, nMZL: nodal marginal zone lymphoma, OR: odds ratio, SNP: single nucleotide polymorphism, VS: versus, HAPMAP: haplotype map

**Supplementary Table S10.** MTHFR prevalence in pSS MALT+ nMZL patients compared to pSS non-MALT patients. Genotypes, OR and p-value for the five genetic models (codominant, dominant, recessive, overdominant and additive) were estimated with SNPstats software (statistically significant if p<0.05).

| **SNPs MTHFR** | **Genotype** | **HAPMAP Database (%)** | **pSS MALT+ nMZL (n=79) n (%)** | **pSS non MALT (n=15) n (%)** | **OR codominant model [95%CI]** | **p-value** | **OR dominant model [95%CI]** | **p-value** | **OR recessive model [95%CI]** | **p-value** | **OR overdominant model [95%CI]** | **p-value** | **OR log-additive model [95%CI]** | **p-value** |
| --- | --- | --- | --- | --- | --- | --- | --- | --- | --- | --- | --- | --- | --- | --- |
|  |  |  |  |  | **CC vs CT vs TT** |  | **(CT-TT) vs CC** |  | **TT vs (CC-CT)** |  | **CT vs (CC-TT)** |  |  |  |
|  | CC | 46.9 | 32 (40.5) | 4 (26.7) | 1.00 | 0.31 | 0.48 [0.13-1.69] | 0.24 | 0.38 [0.10-1.52] | 0.19 | 0.93 [0.30-2.90] | 0.90 | 0.53 [0.24-1.21] | 0.13 |
| **c. 677C>T** | CT | 44.2 | 37 (46.8) | 7 (46.7) | 0.59 [0.15-2.28] |
|  | TT | 8.8 | 10 (12.7) | 4 (26.7) | 0.28 [0.06-1.42] |
|  |  |  |  |  | **AA vs AC vs CC** |  | **(AC-CC) vs AA** |  | **CC vs (AA-AC)** |  | **AC vs (AA-CC)** |  |  |  |
|  | AA | 43.4 | 38 (48.1) | 11 (73.3) | 1.00 | 0.08 | 2.88 [0.82-10.1] | 0.08 | NA [0.00-NA] | 0.06 | 1.72 [0.49-6.07] | 0.39 | 2.83 [0.93-8.61] | 0.04 |
| **c. 1298A>C** | AC | 45.1 | 32 (40.5) | 4 (26.7) | 2.17 [0.61-7.73] |
|  | CC | 11.5 | 9 (11.4) | 0 (0.0) | NA [0.00-NA] |

MTHFR: methylene tetrahydrofolate reductase, pSS: primary Sjogren’s syndrome, MALT: mucosa-associated lymphoid tissue, nMZL: nodal marginal zone lymphoma, OR: odds ratio, SNP: single nucleotide polymorphism, VS: versus, HAPMAP: haplotype map

**Supplementary Table S11.** Prevalence of MTHFR genotypes in pSS patients with DLBCL and pSS patients without lymphoma, adjusted by gender and age. Genotypes, OR and p-value for the five genetic models (codominant, dominant, recessive, overdominant and additive) were estimated with SNPstats software (statistically significant if p<0.05).

| SNPs MTHFR | Genotype | HAPMAP Database (%) | pSS DLBCL (n=12) n (%) | pSS(n=262) n (%) | OR codominant model [95%CI] | p-value | OR dominant model [95%CI] | p-value | OR recessive model [95%CI] | p-value | OR overdominant model [95%CI] | p-value | OR log-additive model [95%CI] | p-value |
| --- | --- | --- | --- | --- | --- | --- | --- | --- | --- | --- | --- | --- | --- | --- |
|  |  |  |  |  | CC vs CT vs TT |  | (CT-TT) vs CC |  | TT vs (CC-CT) |  | CT vs (CC-TT) |  |  |  |
|  | CC | 46.9 | 4 (33.3) | 102 (38.9) | 1.00 | 0.45 | 1.45 [0.42-4.99] | 0.55 | 2.62 [0.64-10.7] | 0.21 | 0.82 [0.25-2.69] | 0.75 | 1.61 [0.69-3.77] | 0.27 |
| c. 677C>T | CT | 44.2 | 5 (41.7) | 119 (45.4) | 1.13 [0.29-4.37] |
|  | TT | 8.8 | 3 (25.0) | 41 (15.7) | 2.80 [0.57-13.84] |
|  |  |  |  |  | AA vs AC vs CC |  | (AC-CC) vs AA |  | CC vs (AA-AC) |  | AC vs (AA-CC) |  |  |  |
|  | AA | 43.4 | 9 (75.0) | 137 (52.3) | 1.00 | 0.15 | 0.37 [0.10-1.40] | 0.12 | 0.00 [0.00-NA] | 0.12 | 0.55 [0.15-2.12] | 0.37 | 0.38 [0.11-1.26] | 0.07 |
| c. 1298A>C | AC | 45.1 | 3 (25.0) | 98 (37.4) | 0.47 [0.12-1.78] |
|  | CC | 11.5 | 0 (0.0) | 27 (10.3) | 0.00 [0.00-NA] |

MTHFR: methylene tetrahydrofolate reductase, pSS: primary Sjogren’s syndrome, DLBCL: diffuse large B-cell lymphoma, OR: odds ratio, SNP: single nucleotide polymorphism, VS: versus, HAPMAP: haplotype map

**Supplementary Table S12.** Prevalence of MTHFR genotypes in pSS patients with DLBCL and healthy Controls, adjusted by gender and age. Genotypes, OR and p-value for the five genetic models (codominant, dominant, recessive, overdominant and additive) were estimated with SNPstats software (statistically significant if p<0.05).

| SNPs MTHFR | Genotype | HAPMAP Database (%) | pSS DLBCL (n=12) n (%) | **Healthy controls (n=600) n (%)** | OR codominant model [95%CI] | p-value | OR dominant model [95%CI] | p-value | OR recessive model [95%CI] | p-value | OR overdominant model [95%CI] | p-value | OR log-additive model [95%CI] | p-value |
| --- | --- | --- | --- | --- | --- | --- | --- | --- | --- | --- | --- | --- | --- | --- |
|  |  |  |  |  | CC vs CT vs TT |  | (CT-TT) vs CC |  | TT vs (CC-CT) |  | CT vs (CC-TT) |  |  |  |
|  | CC | 46.9 | 4 (33.3) | 235 (39.2) | 1.00 | 0.65 | 1.22 [0.36-4.15] | 0.75 | 1.97 [0.50-7.70] | 0.36 | 0.80 [0.25-2.55] | 0.70 | 1.36 [0.60-3.12] | 0.46 |
| c. 677C>T | CT | 44.2 | 5 (41.7) | 291 (48.5) | 1.00 [0.27-3.81] |
|  | TT | 8.8 | 3 (25.0) | 74 (12.3) | 1.98 [0.42-9.35] |
|  |  |  |  |  | AA vs AC vs CC |  | (AC-CC) vs AA |  | CC vs (AA-AC) |  | AC vs (AA-CC) |  |  |  |
|  | AA | 43.4 | 9 (75.0) | 273 (45.5) | 1.00 | 0.11 | 0.32 [0.09-1.23] | 0.08 | 0.00 [0.00-NA] | 0.13 | 0.47 [0.13-1.78] | 0.24 | 0.34 [0.10-1.14] | 0.05 |
| c. 1298A>C | AC | 45.1 | 3 (25.0) | 266 (44.3) | 0.40 [0.10-1.49] |
|  | CC | 11.5 | 0 (0.0) | 61 (10.2) | 0.00 [0.00-NA] |

MTHFR: methylene tetrahydrofolate reductase, pSS: primary Sjogren’s syndrome, DLBCL: diffuse large B-cell lymphoma, OR: odds ratio, SNP: single nucleotide polymorphism, VS: versus, HAPMAP: haplotype map

**Supplementary Table S13.** Prevalence of MTHFR genotypes in pSS patients with DLBCL and pSS patients with MALT lymphoma, adjusted by gender and age. Genotypes, OR and p-value for the five genetic models (codominant, dominant, recessive, overdominant and additive) were estimated with SNPstats software (statistically significant if p<0.05).

| SNPs MTHFR | Genotype | HAPMAP Database (%) | pSS DLBCL (n=12) n (%) | **pSS MALT (n=75) n (%)** | OR codominant model [95%CI] | p-value | OR dominant model [95%CI] | p-value | OR recessive model [95%CI] | p-value | OR overdominant model [95%CI] | p-value | OR log-additive model [95%CI] | p-value |
| --- | --- | --- | --- | --- | --- | --- | --- | --- | --- | --- | --- | --- | --- | --- |
|  |  |  |  |  | CC vs CT vs TT |  | (CT-TT) vs CC |  | TT vs (CC-CT) |  | CT vs (CC-TT) |  |  |  |
|  | CC | 46.9 | 4 (33.3) | 32 (42.7) | 1.00 | 0.52 | 1.73 [0.46-6.53] | 0.41 | 2.38 [0.50-11.2] | 0.29 | 1.01 [0.28-3.64] | 0.98 | 1.65 [0.68-3.97] | 0.26 |
| c. 677C>T | CT | 44.2 | 5 (41.7) | 34 (45.3) | 1.40 [0.33-5.95] |
|  | TT | 8.8 | 3 (25.0) | 9 (12.0) | 2.83 [0.50-16.2] |
|  |  |  |  |  | AA vs AC vs CC |  | (AC-CC) vs AA |  | CC vs (AA-AC) |  | AC vs (AA-CC) |  |  |  |
|  | AA | 43.4 | 9 (75.0) | 34 (45.3) | 1.00 | 0.08 | 0.29 [0.07-1.17] | 0.07 | 0.00 [0.00-NA] | 0.08 | 0.49 [0.12-2.03] | 0.31 | 0.30 [0.08-1.08] | 0.04 |
| c. 1298A>C | AC | 45.1 | 3 (25.0) | 32 (42.7) | 0.38 [0.09-1.59] |
|  | CC | 11.5 | 0 (0.0) | 9 (12.0) | 0.00 [0.00-NA] |

MTHFR: methylene tetrahydrofolate reductase, pSS: primary Sjogren’s syndrome, DLBCL: diffuse large B-cell lymphoma, MALT: mucosa-associated lymphoid tissue, OR: odds ratio, SNP: single nucleotide polymorphism, VS: versus, HAPMAP: haplotype map
